# Esquirol on Insanity

**Published:** 1840

**Authors:** 


### *On what Cerebral Alterations does Insanity depend?*

This important question is almost answered by an admission of ignorance on the part of M. Esquirol. He offers, however, a general summary of the results hitherto obtained in this inquiry.

1. Malformations of the cranium are only observed in imbeciles, idiots and cretins.

2. Organic lesions of the encephalon or its envelopes have only been observed in those whose insanity was combined with paralysis, convulsions, epilepsy. The lesions belonged to the complication, not to the insanity.

3. The sanguineous or serous effusions—the injections or infiltrations—the thickening of the membranes—the ramollissement, induration, tumors of the encephalon, &c.—all these alterations indicate the causes or the effects of insanity, or rather the effects of its complication with the malady which proved fatal to the patient.

4. The alterations of the thorax, abdomen, pelvic cavity, are evidently independent of insanity. Yet they point out the organ whose alterations first disturbed the brain.

5. All the organic lesions found in the insane have been found in those who have never exhibited insanity.

6. In many instances, examination of the body after death, has led to the discovery of no alteration, although the insanity had existed for years.

7. Pathological anatomy shews us each part of the encephalon altered, or destroyed, without the occurrence of insanity.

8. From all these data we may conclude, *that* the immediate cause of insanity is beyond our means of investigation; *that* the disorder depends on some unknown modification of the brain; and *that* it does not always originate in the brain, but in some of the seats of sensibility placed in the various regions of the body. This may seem discouraging, but if true, should be well known. Perhaps, as M. Esquirol observes, an exact acquaintance with the immediate cause of insanity would not help us much in the treatment of it. Probably, if we were exactly acquainted with the subtle condition of the nervous system which produces pain, we should not be able to lull it one whit better than we do.

*Prognosis of Insanity.*—Imbecility and idiotcy are never cured

Monomania and melancholia, when recent, accidental, and independent of organic lesion, are curable.

Mania is more curable than monomania and melancholia.

Acute dementia is sometimes cured—chronic dementia is rarely cured—and senile dementia never.

Hereditary insanity is curable, but relapses are more probable than in accidental insanity.

Chronic insanity is with difficulty cured, especially after the second year; the difficulty is augmented in proportion to the length of time the causes have operated prior to the occurrence of the malady.

However long the malady may have existed, recovery may take place while palpable derangements of the corporeal functions exist.

If the moral causes have been sudden in their operation, the prognosis is more favorable, than if they have acted gradually.

If insanity has been produced by excess of study, the prognosis is unfavorable, especially when errors of regimen have been combined with the over-exertion of the mind.

Insanity dependent on religious excitement, or on pride, is seldom cured.

Insanity kept up by hallucinations is very difficult of removal.

When the insane are well aware of their condition, yet are not promptly cured, the prognosis is unfavorable.

When the insane recover their bodily health, yet evince no progress towards mental restoration, we must not be sanguine of their recovery.

When the senses of the insane are so enfeebled that they can look upon the sun without uneasiness, and have lost smell, and taste, &c., they are incurable.

Insanity is incurable when the consequence of scurvy, or epilepsy. Its complication with these diseases and with paralysis is invariably fatal.

Such is the summary of data for prognosis, with which M. Esquirol presents us.

We pass to the *treatment.*

The leading objects are to calm their passions—to remove the mental infirmity or aberration—to remove the physical disturbance. Each case must be studied—the causes of the disease investigated—the character of the patient determined. There is no specifiic treatment for insanity. As the causes of the malady are moral or physical, the means of treatment must be physical and moral too.

*Esquirol in Med. Chirug. Review.*

